# Relationship between the amplitudes of cerebral blood flow velocity and intracranial pressure using linear and non-linear approach

**DOI:** 10.1007/s10877-024-01243-1

**Published:** 2024-12-10

**Authors:** Adam I. Pelah, Monika Najdek, Marek Czosnyka, Agnieszka Uryga

**Affiliations:** 1 Division of Neurosurgery, Department of Clinical Neurosciences, Addenbrooke’s Hospital, University of Cambridge, Cambridge, UK; 2 Department of Biomedical Engineering, Faculty of Fundamental Problems of Technology, Wroclaw University of Science and Technology, Wroclaw, Poland

**Keywords:** Intracranial pressure, Cerebral blood flow velocity, Cerebral blood volume, Traumatic brain injury

## Abstract

Intracranial pressure (ICP), cerebral blood flow and volume are affected by craniospinal elasticity and cerebrospinal fluid dynamics, interacting in complex, nonlinear ways. Traumatic brain injury (TBI) may significantly alter this relationship. This retrospective study investigated the relationship between the vascular and parenchymal intracranial compartments by analysing two amplitudes: cerebral blood flow velocity (AmpCBFV) and ICP (AMP) during hypocapnia manoeuvre in TBI patients. Twenty-nine TBI patients hospitalised at Addenbrooke’s Hospital, whose ICP and CBFV were monitored during mild hypocapnia, were included. A linear metric of the relationship was defined as a moving-window correlation (R) between AmpCBFV and AMP, named RAMP. Nonlinear metrics were based on the Joint Symbolical Analysis (JSA) algorithm, which transforms AmpCBFV and AMP into sequences of symbols (‘words’) using a binary scheme with word lengths of three. The mean AmpCBFV and AMP were not significantly correlated at baseline (r = − 0.10) or during hypocapnia (r = − 0.19). However, the RAMP index was significantly higher at baseline (0.64 ± 0.04) compared to hypocapnia (0.57 ± 0.04, p = 0.035). The relative frequency of symmetrical word types (JSA_sym_) describing the AmpCBFV–AMP interaction decreased during hypocapnia (0.35 ± 0.30) compared to baseline (0.44 ± 0.030; p = 0.004). Our results indicate that while the grouped-averaged AmpCBFV and AMP were not significantly correlated, either at baseline or during hypocapnia, significant changes were observed when using RAMP and JSA indices. Further validation of these new parameters, which reflect the association between the vascular and parenchymal intracranial compartments, is needed in a larger cohort.

## Introduction

Intracranial pathologies are among the leading causes of death and morbidity worldwide. About 50–60 million people sustain traumatic brain injury (TBI) each year, where young people constitute a significant percentage of patients [1–3]. A better understanding of the pathological changes in cerebral circulation and hemodynamics using high-frequency multimodal recordings could facilitate improved care.

The cranium, a rigid structure, contains three main components: the brain, cerebrospinal fluid (CSF) and blood. Following a head injury, intracranial mass lesions, brain swelling, CSF circulation disorders, intracranial haemorrhage, or more diffuse pathological processes can lead to an increase in intracranial pressure (ICP) [4]. Increased ICP can, in turn, restrict cerebral blood flow (CBF) and result in ischaemia. The relationship between ICP and intracranial volume is represented by the non-linear pressure–volume curve [5], which illustrated intracranial compliance (ICC). Over the decades, ICC has been extensively studied for its potential to provide a more accurate understanding of brain deterioration [6, 7].

The linear correlation between the ICP pulse amplitude (AMP) and mean ICP serves as the compensatory reserve index (RAP) [8]. Its utility as a measure of compensatory reserve and ICC has been studied in previous studies [4, 9]. However, research has shown that in patients with head trauma, metrics of the pressure–volume reserve that rely solely on ICP do not consistently reflect ICC [10]. For example, Kiening et al. found that episodes of raised ICP > 20 mm Hg were associated with reduced CSF space compliance in only 40% of patients [11]. Furthermore, recent studies indicate that RAP measurements can be affected by baseline effect errors (BEEs) [12]—spontaneous shifts in baseline pressure that occur during continuous ICP monitoring, first identified by Eide [13]. This suggests a need to explore alternative metrics.

Although AMP has been a focal point of research and clinical work in recent years, a pulsatile component with a distinct morphology is also present in the cerebral blood flow velocity (CBFV) signal, which is measured using transcranial Doppler ultrasonography (TCD). Simultaneous use of transcranial Doppler ultrasonography (TCD) and an intraparenchymal probe allows monitoring of two different intracranial compartments: the vascular and parenchymal compartments. While the amplitude of the CBFV (AmpCBFV) has been utilised in indices such as the pulsatility index, the link between it and AMP has yet to be established. The amplitude of the CBFV controls the pulse amplitude of the cerebral arterial blood volume, where the link is time integration. Then the amplitude of cerebral arterial blood volume controls AMP, where the link is the shape of a pressure‒volume curve. Therefore, analysis of its dynamics may elucidate pressure‒volume compliance, especially during a stimulus such as moderate hypocapnia. Moderate, short-term hypocapnia can cause vasoconstriction, leading to a reduction in cerebral blood volume (CBV) on the arterial side and a subsequent decrease in ICP [14, 15]. The effects of hypocapnia on decreased CBFV reflect corresponding changes in CBV [15].

In addition to the need for complex analysis of intracranial compartments, there is also a lack of methodology to describe the relationships between biosignals, which may vary in time. Correlation and correlation-coefficient-based indices are important tools for analysing the similarity between time series and is easy to interpret. However, such a simple approach may lead to the ‘leakage’ of crucial information about temporal changes or synchronisation/desynchronization periods, as the relationship between two signals may not be linear. An interesting nonlinear concept is joint symbolical analysis (JSA), a method that has long been recognised in cardiology for characterising the association between cardiac and respiratory rhythms [16–18]. Conceptually, symbolic dynamics refers to binary symbolisation, which provides a very compact representation of variability in the time series [19].

In this study, we aimed to analyse the relationship between AmpCBFV and AMP during hypocapnia in a TBI cohort of patients. We hypothesise that using linear and nonlinear methods may better reflect the relationship between the vascular and parenchymal intracranial compartments.

## Materials and methods

### Study design

This retrospective study included head-injured patients hospitalised at Addenbrooke’s Hospital (Cambridge, UK) between March 2001 and February 2002. The data were collected in a prospective study by Steiner et al. [20] and were reanalysed in this study. Analysing the data recorded from past studies was motivated by new findings about the association between the CBFV waveform and changes in the morphology of the ICP [21]. The collection of these data was prospectively considered by the multidisciplinary NCCU Users Group, and it was agreed that because the assessment of CO_2_ reactivity was part of normal clinical management and since no patient confidentiality issues were involved, formal informed consent was not needed. These anonymized data are subsequently made available for future research. The data curation process adheres to the research integrity guidelines outlined in the UK Health Departments' (2011) Governance Arrangements for Research Ethics Committees. The inclusion criteria were cerebral perfusion pressure (CPP) ≥ 70 mm Hg, a normocapnic baseline with end-tidal CO_2_ (EtCO_2_) and age ≥ 16 years. The exclusion criteria were respiratory failure, a baseline partial pressure of carbon dioxide (paCO_2_) < 4.30 kPa, failure to obtain satisfactory bilateral transcranial Doppler signals and craniotomy. Patients were analgo-sedated with propofol (2–5 mg/kg/h) and fentanyl (1–2 mcg/kg/h). All patients were mechanically ventilated and supported by norepinephrine or mannitol if necessary to maintain the CPP/ICP level, according to current guidelines, which recommend a threshold of 20–25 mmHg above which an increased ICP should be treated [22]. Data were collected during routine determination of CO_2_ reactivity. After baseline data were recorded for 20 min and a baseline value for paCO_2_ was obtained, the minute volume of the ventilator was increased by 15–20%. If, owing to this intervention, the standard treatment guidelines (paCO_2_ > 3.5 kPa and/or jugular bulb venous oxygen saturation (SjvO_2_) > 55%) were exceeded, the protocol was abandoned. After an initial stabilisation period of 10 min, the end-tidal CO_2_ was kept stable, and the data were recorded again for 20 min. PaCO_2_ was measured at the middle of this stable phase. After CO_2_ reactivity testing had been completed, PaCO2 was slowly adjusted to the level that the responsible physician deemed appropriate. Importantly, there were no pharmacologic manipulations of the ICP or ABP during the examination protocol, and the analgo-sedation was kept unchanged during the recorded periods of baseline and hypocapnia.

### Signal recording

CBFV was measured using a transcranial Doppler ultrasonography (TCD) with 2-MHz Doppler probes insonating the middle cerebral arteries (MCAs) bilaterally (Multi Dop X4, DWL Elektronische Systeme, Sipplingen, Germany). The intraparenchymal ICP (Codman MicroSensors ICP Transducer, Codman & Shurtle, Raynham, MA, USA) was monitored via a catheter inserted into the brain tissue. Arterial blood pressure (ABP) was measured using a radial catheter (ABP; Edwards Lifesciences, Irvine, CA, USA). The monitoring included mainstream EtCO_2_ (Marquette Solar 8000 M, GE Medical Systems, UK) and arterial CO_2_ partial pressure (PaCO_2_) using an AVL Omni blood gas analyser (AVL Omni, Graz, Austria). The signals were acquired with a sampling frequency of 30 Hz using an analogue–digital converter (DT9801 and DT9803, Data Translation, Marlboro, MA, USA) and recorded using ICM + software (Cambridge Enterprise, Cambridge, UK, http://icmplus.neurosurg.cam.ac.uk).

### Signal processing and parameter definitions

**Fig. 1:**
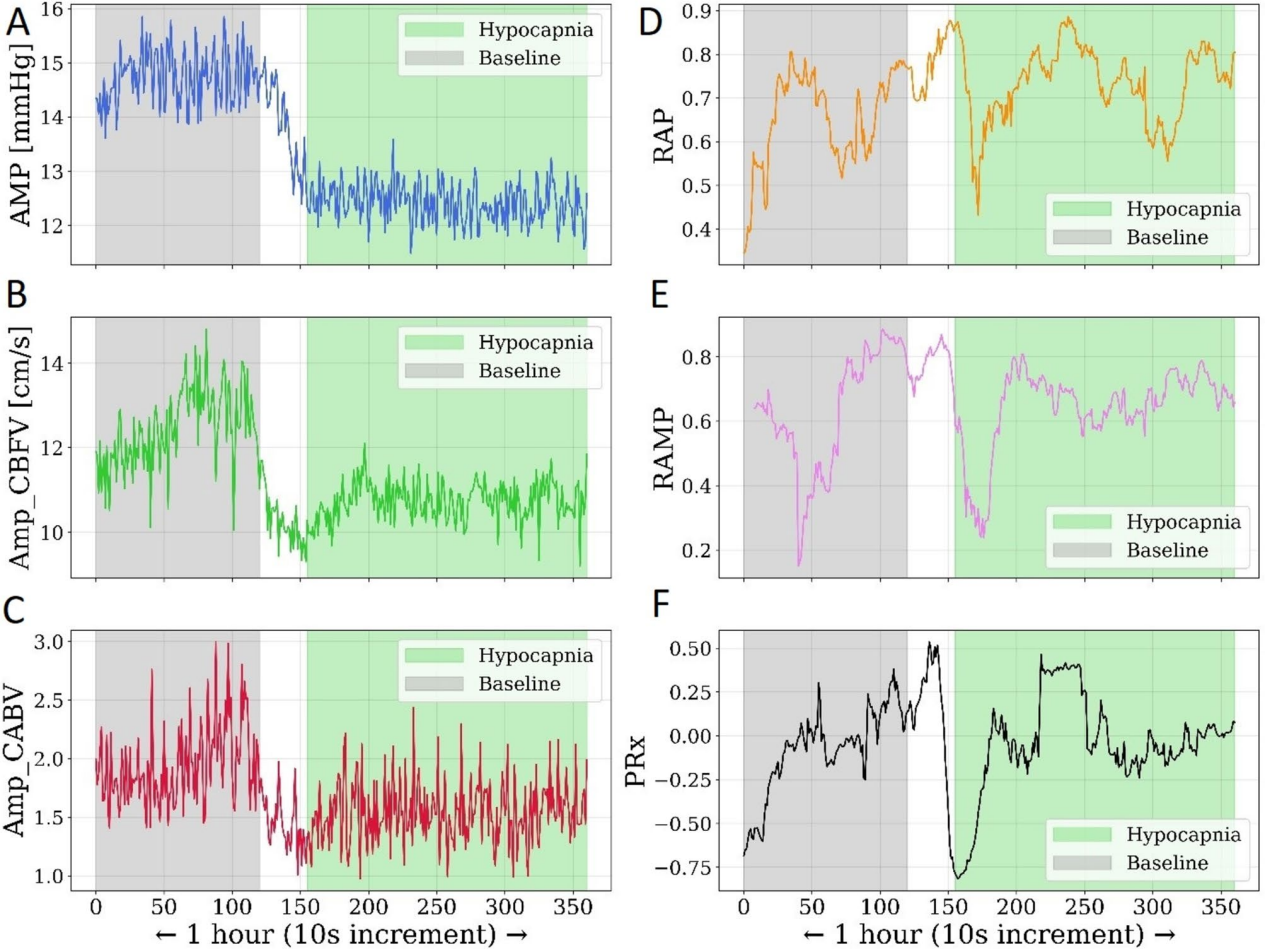
An exemplary time trend of (*left panels*): estimated neuroparameters averaging in the 10-s time window: pulse amplitude of **A** ICP (AMP), **B** CBFV (AmpCBFV), **C** C_a_BV (AmpC_a_BV); (*right panel*): correlation between **D** ICP and AMP (RAP index), **E**. AmpCBFV and AMP (RAMP index), **F** ICP and MAP (PRx index) at baseline (*grey part*) and during the hypocapnia plateau phase (*green part*) in patient with traumatic brain injury. ICP, intracranial pressure; CBFV, cerebral blood flow velocity; C_a_BV, cerebral arterial blood volume, MAP-mean arterial pressure

Data analysis was performed using Intensive Care Monitor (ICM +) software (Cambridge University, Cambridge, UK). The heart rate (HR) was estimated as the frequency of the fundamental harmonic of the ABP signal in the frequency range of 0.66–3.00 Hz. The algorithm used to calculate the normalised cerebral arterial blood volume (C_a_BV) was discussed in detail in a previous paper by our group [46]. Here, we provide only a brief interpretation. The changes in cerebral arterial blood volume (C_a_BV) using the constant flow forward (CFF) model during a cardiac cycle can be calculated as the time integral of the difference between the pulsatile CBFV and the mean CBFV [23–25]. Owing to the unknown cross-sectional area of the insonated vessel, it is expressed as normalised C_a_BV, i.e., the pulsatile fraction of arterial blood volume per 1 cm^2^ of cross-sectional area of the vessel. The ICP waveform was decomposed using a fast Fourier transform (FFT) algorithm. This operation allows for oscillations of different frequencies (such as the fundamental component corresponding to the cardiac cycle) to be analyzed separately because in the time domain they are overlaid with each other. AMP was expressed as the amplitude of the fundamental harmonic of the ICP pulse waveform within the range containing the heart rate frequency of adult humans (40–180 bpm, corresponding to 0.67–3 Hz) by using spectral analysis based on FFT [26, 27]. Similarly, pulse amplitude of the CBFV (AmpCBFV), and pulse amplitude of the C_a_BV (AmpC_a_BV) were calculated as the amplitude of the fundamental harmonic of the respective signal in the frequency range of 0.66–3.00 Hz. The RAP was calculated as the moving correlation coefficient between the amplitude of the fundamental harmonic of the ICP pulse waves and the mean ICP from a 5-min period with an update over 10 s [9]. The pressure reactivity index (PRx) was calculated as the moving correlation coefficient between the ICP and mean ABP from 5 min with an update over 10 s [28, 29]. The mean velocity index (Mxa) was calculated as the moving correlation coefficient between the CBFV and mean ABP from 5 min, with an update over 10 s [30]. CPP was defined as the difference between the mean ABP and the mean ICP. At first, all artefacts (involving abnormal values or sharp spikes) were identified and manually removed from raw data files in the ICM + software before proceeding with further analysis. Then, as all variables were averaged over 10-s intervals, any remaining fluctuations were smoothed to ensure consistency and accuracy in the data before proceeding with further analysis. The exemplary time trends of the estimated neuroparameters are presented in Fig. 1.

### The index of cerebral pressure‒flow amplitude association (RAMP)

**Fig. 2:**
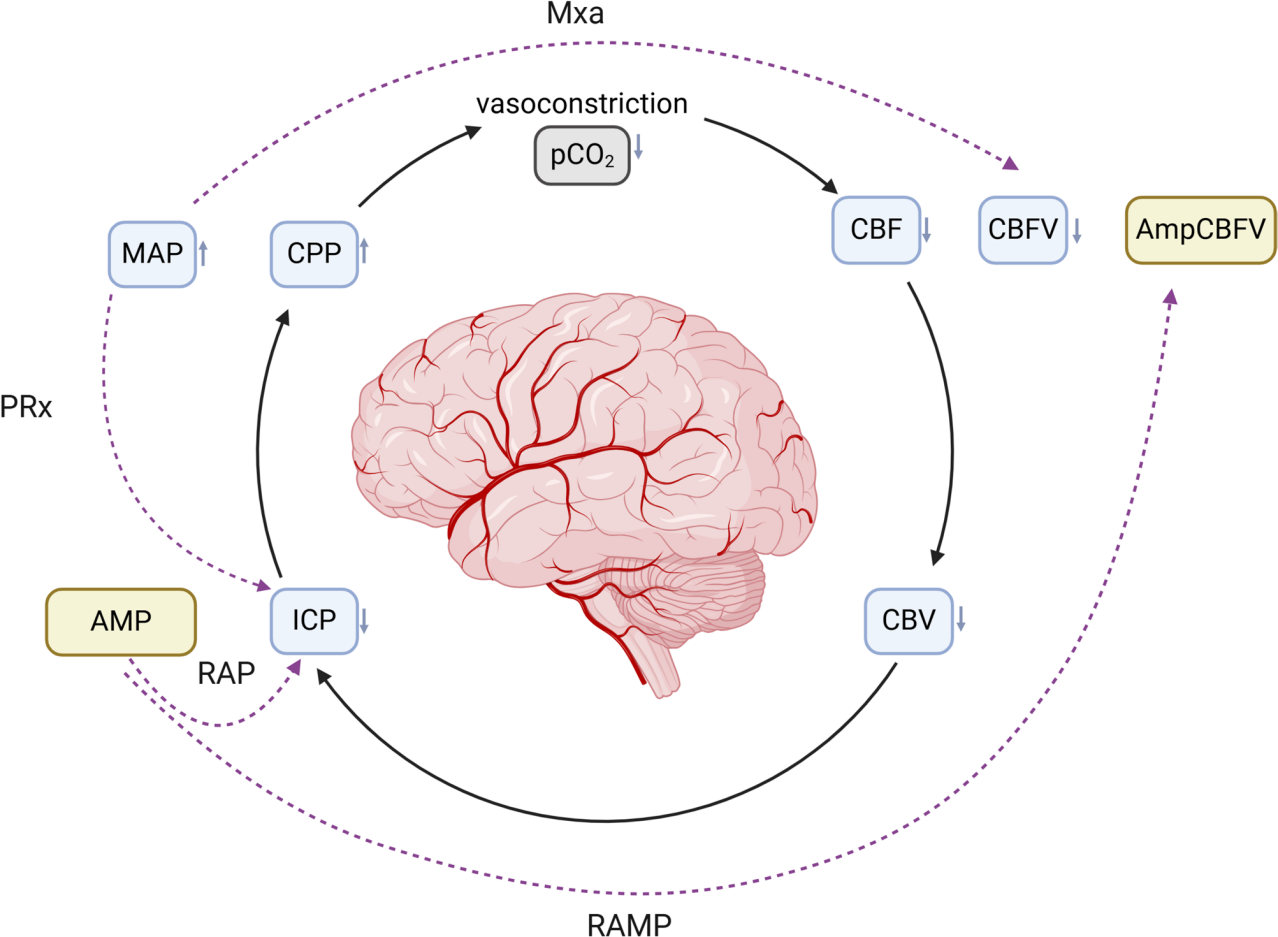
A decrease in partial pressure of carbon dioxide (paCO₂) acts as the initial trigger for cerebral vasoconstriction. As a result, cerebral blood flow (CBF), which could be proxy by cerebral blood flow velocity (CBFV), and cerebral blood volume (CBV) decreases. Consequently, intracranial pressure (ICP) begins to decrease due to the reduction in CBV and CBF. In response, mean arterial pressure (MAP) and cerebral perfusion pressure (CPP) increase. The pressure reactivity index (PRx) describes the relationship between MAP and ICP, while the cerebral autoregulatory index of mean velocity (Mxa) reflects the relationship between MAP and CBFV. The linear correlation between ICP pulse amplitude (AMP) and mean ICP serves as the compensatory reserve index (RAP). The proposed new index, representing the association between the vascular and parenchymal intracranial compartments, is termed RAMP and describes the relationship between AMP and the amplitude of CBFV (AmpCBFV)

In this study, we propose a new index of the vascular and parenchymal intracranial compartment, named RAMP (*correlation *(*R*)* of amplitudes *(*AMP*)). The RAMP was calculated as the moving correlation coefficient between the amplitude of the fundamental harmonic of the CBFV pulse waves and the amplitude of the fundamental harmonic of the ICP pulse waves from a 5-min period with an update over 10 s. The concept and definition of RAMP are presented in Fig. 2.

### Joint symbolical analysis

We adapted JSA method, which was previously used to capture cardiovascular dynamics to track synchronisation and variability in CBFV and ICP amplitudes. Given the time series of AmpCBFV ($x_{n}^{AmpCBFV}$ and AMP ($x_{n}^{AMP}$, we can consider a bivariate vector *X* of length *n*, where x $\in R$:

1 $$X = \left\{ {\left[ {x_{n}^{AmpCBFV} ,x_{n}^{AMP} } \right]^{T} } \right\}$$

The formula proposed by Baumert [16, 19] can be transformed into a bivariate symbol sequence *S,* where s $\in \left\{ {0,1} \right\}$:

2 $$S = \left\{ {\left[ {s_{n}^{AmpCBFV} ,s_{n}^{AMP} } \right]^{T} } \right\}$$

Hence, $s_{n}^{AmpCBFV}$ is defined as 1 when $x_{n}^{AmpCBFV} - x_{n + 1}^{AmpCBFV} < l^{AmpCBFV}$, and $s_{n}^{AMP}$ is defined as 1 when $x_{n}^{AMP} - x_{n + 1}^{AMP} < l^{AMP}$. Otherwise, $s_{n}^{AmpCBFV}$ and $s_{n}^{AMP}$ are defined as 0. In the following, the threshold values are set to zero ($l^{AmpCBFV}$ = 0, $l^{AMP}$ = 0). The words of length three result in 64 different word types, which provide a statistically sufficient representation of dynamics within 30 min of beat-to-beat time series data [19]. The relative frequency of each of the 8 × 8 combinations of binary symbolic sequences $s_{n}^{AmpCBFV}$ and $s_{n}^{AMP}$ obtained from the bivariate time series *Z* can be written as a word distribution matrix *W,* from word type [000,000]^T^ to [111,111]^T^:

3 $$W = \left[ {\begin{array}{*{20}c} {AmpCBFV_{000,} } \\ {AmpCBFV_{111,} } \\ \end{array} \vdots \begin{array}{*{20}c} {AMP_{000,} } \\ {AMP_{000,} } \\ \end{array} \begin{array}{*{20}c} \cdots \\ \ddots \\ \cdots \\ \end{array} \begin{array}{*{20}c} {AmpCBFV_{000,} } \\ {AmpCBFV_{111,} } \\ \end{array} \vdots \begin{array}{*{20}c} {AMP_{111} } \\ {AMP_{111,} } \\ \end{array} } \right]$$

Based on the *W* matrix, the relative frequency of symmetrical word types (JSA_sym_) and the opposite patterns (JSA_diam_) was defined accordingly [19]:

4 $$JSA_{sym} = \mathop \sum \limits_{j = k = 1}^{8} W_{j,k}$$

5 $$JSA_{diam} = \mathop \sum \limits_{j = k = 1}^{8} W_{j,9 - k}$$

**Fig. 3:**
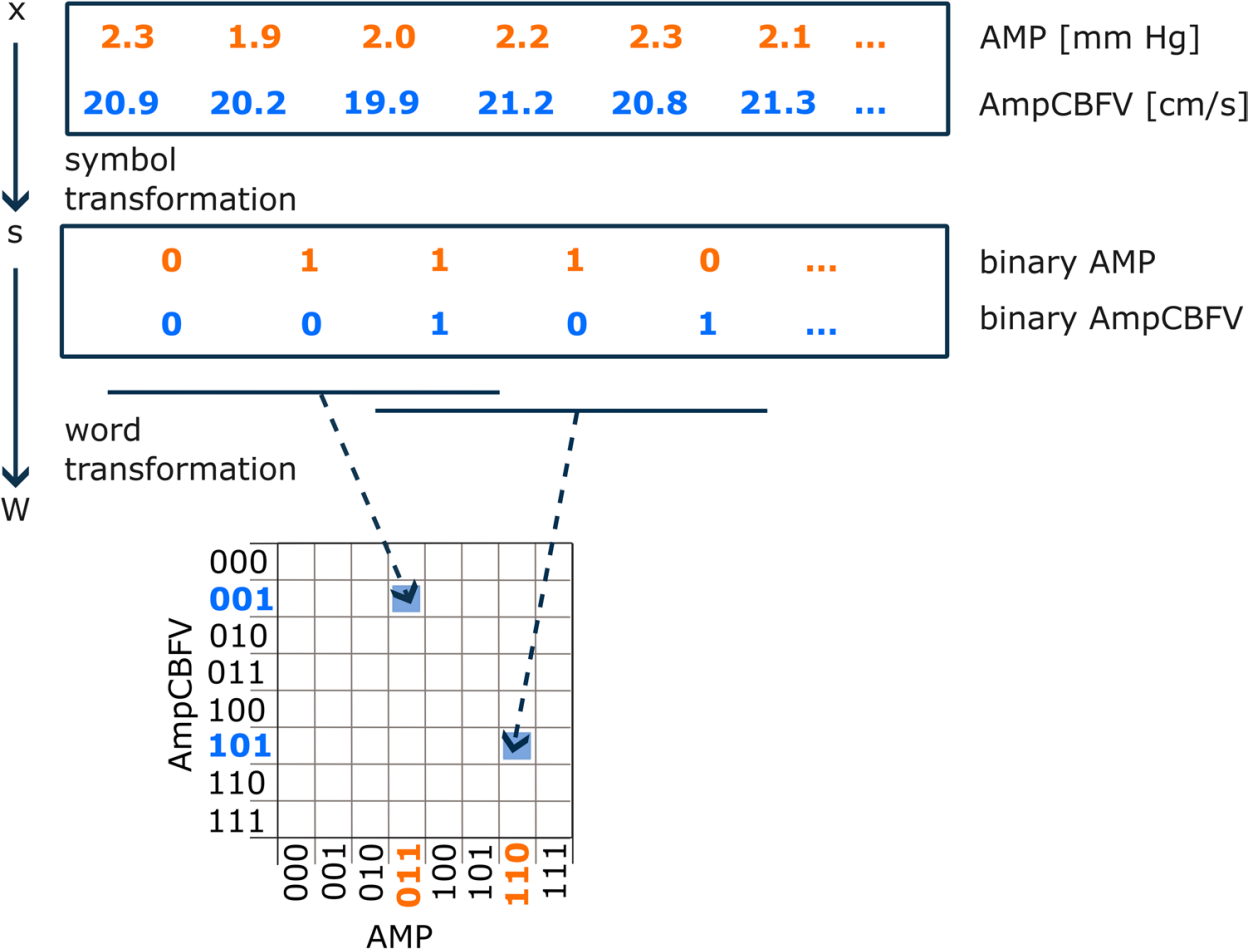
Schematic representation of the joint symbolic analysis of pulse amplitude of intracranial pressure (AMP) and pulse amplitude of cerebral blood flow velocity (AmpCBFV) time series using a binary symbolisation procedure. Vector x of beat-to-beat changes in AMP and AmpCBFV, respectively, was transformed to vector *s* of sequences of 1 (increases) and 0 (decreased or no changes) and word distribution density matrix *W*

A schematic transformation of vector *X*, containing bivariate AmpCBFV and AMP samples, into symbol vector *S* and word distribution matrix W is presented in Fig. 3.

### Statistical methods

The normality of the data distributions was tested using the Kolmogorov–Smirnov test with Lilliefors correction. A dependent-sample t-test was run to determine if the average neuromonitoring parameters changed during hypocapnia. The relationships between two neuromonitoring parameters (AmpCBFV and AMP) were examined using the Pearson correlation coefficient (r). Student’s t-test was used to compare two independent groups (regarding thresholds for ICP and PRx), where the assumption of homogeneity of variance was tested with Levene’s test. The data are presented as the means ± standard error unless otherwise indicated. The level of statistical significance was set as 0.05. All analyses were performed in Python.

## Results

### Impact of hypocapnia on neuromonitoring parameters

Twenty-nine TBI patients (median age 39 years, 5 females) with severe injuries (mean Glasgow Coma Scale score of 6 ± 3) were included. The mean values of the physiological parameters are presented in Table 1. Both ICP and CBFV, as well as their respective amplitudes, decreased significantly during hypocapnia (p < 0.001 for all) (see Table 1). The AmpC_a_BV decreased significantly (p < 0.001). During hypocapnia, no significant changes in cerebral autoregulation were found. The RAP decreased during hypocapnia (p = 0.039).

**Table 1:** Mean values of physiological parameters and estimated neuroparameters at baseline and during the plateau phase of hypocapnia

Parameter	Baseline	Hypocapnia	p value
ABP [mmHg]	96 ± 2	99 ± 2	0.220
HR [bpm]	78.5 ± 3.0	78.9 ± 3.0	0.698
ICP [mmHg]	16 ± 1	12 ± 1	**< 0.001**
AMP [mmHg]	1.9 ± 0.3	1.4 ± 0.3	**< 0.001**
CPP [mmHg]	81 ± 2	87 ± 2	**0.003**
CBFV [cm/s]	75 ± 6	60 ± 5	**< 0.001**
AmpCBFV [cm/s]	21.4 ± 1.5	18.8 ± 1.4	**< 0.001**
AmpC_a_BV [cm]	3.0 ± 0.2	2.5 ± 0.2	**< 0.001**
RAMP	0.64 ± 0.04	0.57 ± 0.04	**0.035**
RAP	0.45 ± 0.07	0.36 ± 0.06	**0.039**
PRx	0.01 ± 0.04	− 0.02 ± 0.03	0.460
Mxa	− 0.02 ± − 0.06	− 0.04 ± − 0.04	0.610
JSA_sym_	0.44 ± 0.03	0.35 ± 0.03	**0.004**
JSA_dia_	0.04 ± 0.01	0.06 ± 0.01	0.059

The sample size was 29 for each record. The data are presented as the means with standard errors. Significant differences are marked in bold

ABP, arterial blood pressure; HR, heart rate; ICP, intracranial pressure; CPP, cerebral perfusion pressure; CBFV, cerebral blood flow velocity; AMP, intracranial pressure pulse amplitude; AmpCBFV, pulse amplitude of cerebral blood flow velocity; AmpC_a_BV—amplitude of cerebral arterial blood volume; RAMP, correlation between AmpCBFV and AMP; RAP, compensatory reserve; PRx, pressure reactivity index; Mxa, mean velocity index; JSA_sym_, joint symbolic analysis (symmetrical); JSA_dia_, joint symbolic analysis (diagonal)

### Relationship between AmpCBFV and AMP using linear method

**Fig. 4:**
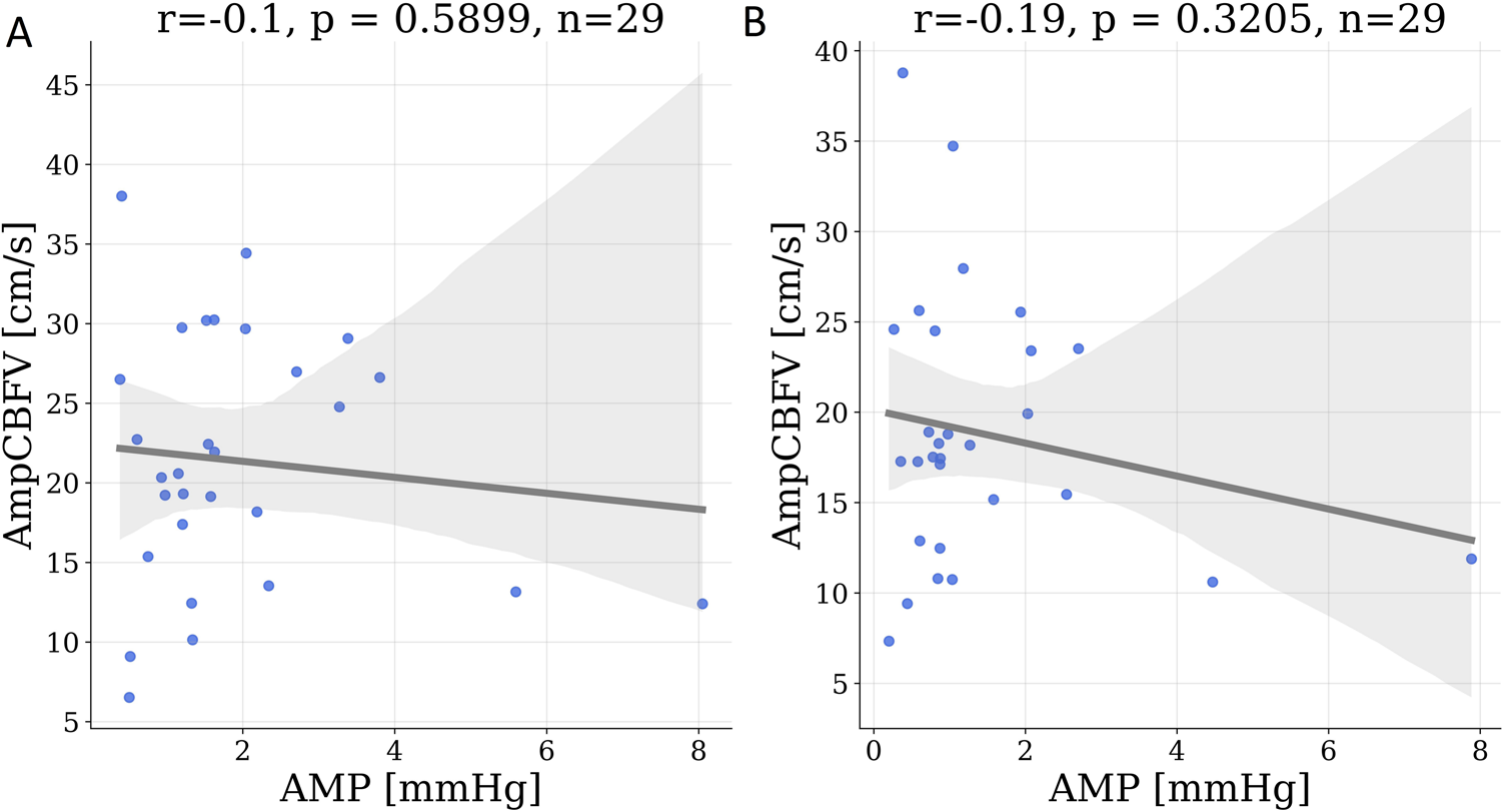
Relationship between the pulse amplitude of intracranial pressure (AMP) and the pulse amplitude of cerebral blood flow velocity (AmpCBFV) at **A** baseline and **B** hypocapnia, as illustrated using scatter plots

The mean AmpCBFV and AMP were not significantly correlated with each other, both at baseline (r = − 0.1, p = 0.59; Fig. 4A) and during the hypocapnia phase (r = − 0.19, p = 0.32; Fig. 4B). The RAMP was significantly higher during the baseline phase (0.64 ± 0.04) than during the hypocapnia phase (0.57 ± 0.04, p = 0.035).

### Relationship between AmpCBFV and AMP using non-linear method

**Fig. 5:**
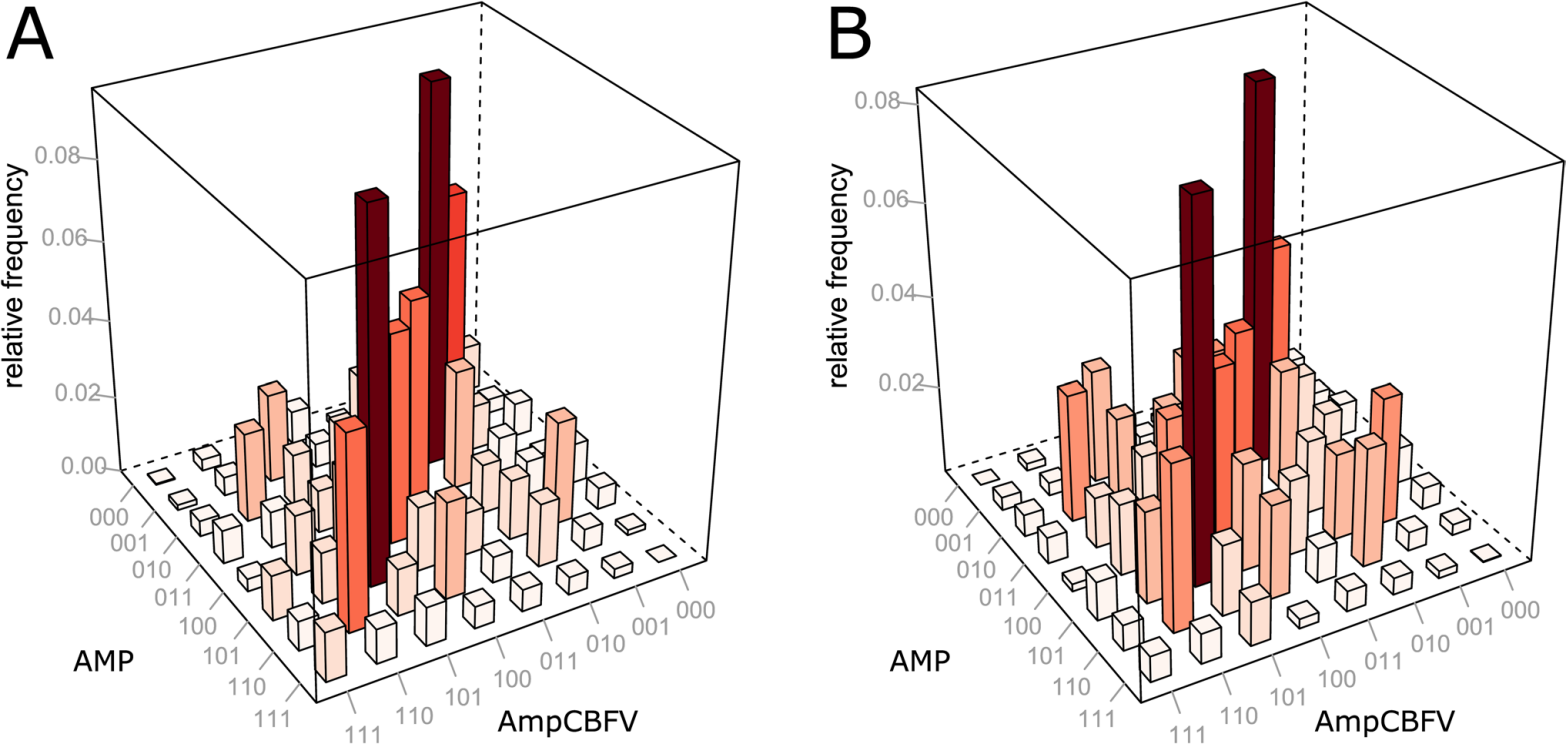
Word distribution matrices of joint symbolic dynamics during the hypocapnia procedure for **A** the baseline and **B** hypocapnia. AMP, pulse amplitude of intracranial pressure, AmpCBFV, pulse amplitude of cerebral blood flow velocity

The mean values of JSA_sym_ and JSA_dia_ are presented in Table 1. We observed that JSA_sym_ significantly decreased during hypocapnia (0.35 ± 0.30) compared with that at baseline (0.44 ± 0.03; p = 0.004). Bar plots presenting the frequency distribution of word types illustrating the time course-graining synchronisation between AmpCBFV and AMP are presented in Fig. 5A (baseline) and Fig. 5B (hypocapnia). A shift in values from symmetrical towards the opposite direction is observed during hypocapnia.

### Relationship between AmpCBFV and AMP *vs.* ICP and cerebral autoregulation thresholds

**Fig. 6:**
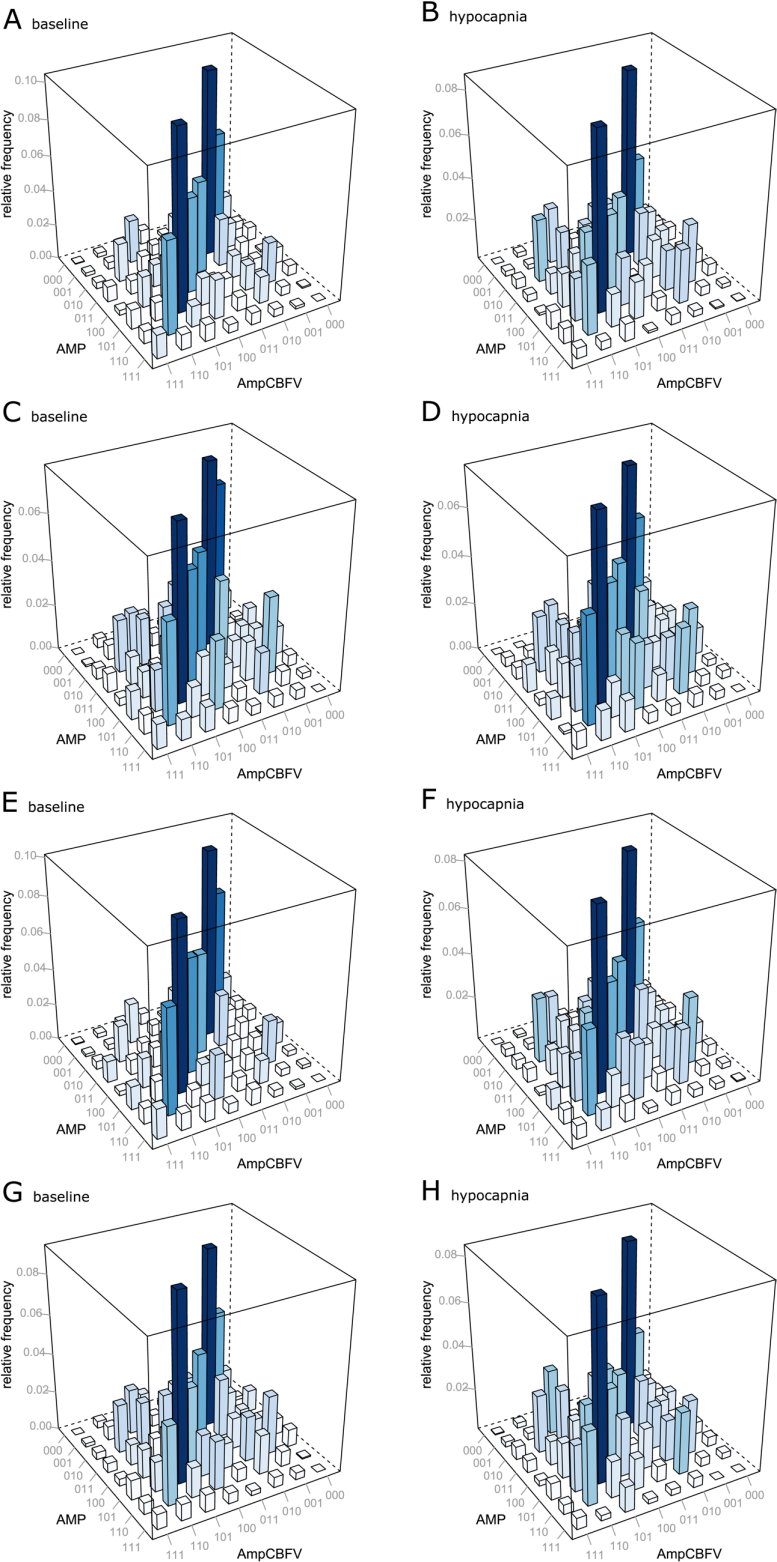
Word distribution matrices of joint symbolic analysis at baseline and during hypocapnia regarding. **A**, **B** intracranial pressure (ICP) values less than 20 mmHg **C**, **D** ICP values greater than or equal to 20 mmHg, **E**, **F** pressure reactivity index (PRx) values less than 0, **G**, **H** PRx values greater than or equal to 0. AMP, pulse amplitude of intracranial pressure; AmpCBFV, pulse amplitude of cerebral blood flow velocity

The mean values of neuroparameters at baseline and during hypocapnia *vs*. thresholds for increased ICP and impaired cerebral autoregulation are presented in Table 2. We found that in patients with an ICP < 20 mmHg (normal ICP), JSA_sym_ significantly decreased (p = 0.016), whereas JSA_dia_ increased (p = 0.038) during hypocapnia, reflecting a shift in values from symmetrical to the opposite direction. With respect to cerebral autoregulation, in patients with a PRx < 0 (preserve autoregulation), AMP significantly decreased (p = 0.023), JSA_sym_ decreased (p = 0.022), and JSA_dia_ increased (p = 0.016) during hypocapnia. However, when Mxa was used, we found that in patients with Mxa > 0 (impaired autoregulation) JSA_sym_ decreased (p = 0.025), Table 2. The word distribution matrices for AMP-AmpCBFV at baseline and during hypocapnia *vs*. ICP and cerebral autoregulation thresholds are presented in Fig. 6.

**Table 2:** Mean values of estimated neuroparameters at baseline and during the plateau phase of hypocapnia regarding the thresholding values of intracranial pressure (ICP) and cerebral autoregulation derived via the pressure reactivity index (PRx) and mean velocity index (Mxa)

	ICP < 20 mmHg (n = 21)			ICP ≥ 20 mm Hg (n = 8)		
	Baseline	Hypocapnia	p value	Baseline	Hypocapnia	p value
AMP [mmHg]	1.42 ± 0.20	0.99 ± 0.10	0.056	3.26 ± 0.90	2.51 ± 0.90	0.561
AmpCBFV [cm/s]	20.91 ± 2.00	18.33 ± 2.00	0.287	22.61 ± 3.00	20.13 ± 3.00	0.534
RAMP [a.u.]	0.63 ± 0.06	0.56 ± 0.05	0.371	0.69 ± 0.06	0.58 ± 0.07	0.267
JSA_sym_ [a.u.]	0.45 ± 0.04	0.36 ± 0.03	**0.016**	0.40 ± 0.08	0.33 ± 0.05	0.153
JSA_dia_ [a.u.]	0.04 ± 0.01	0.06 ± 0.01	**0.038**	0.05 ± 0.03	0.06 ± 0.02	0.584

	PRx < 0 (n = 17)			PRx ≥ 0 (n = 12)		
	Baseline	Hypocapnia	p value	Baseline	Hypocapnia	p value
AMP [mmHg]	1.47 ± 0.20	0.91 ± 0.10	**0.023**	2.57 ± 0.60	2.11 ± 0.60	0.616
AmpCBFV [cm/s]	21.37 ± 2.00	18.08 ± 1.00	0.119	21.40 ± 3.00	19.89 ± 3.00	0.708
RAMP [a.u.]	0.67 ± 0.05	0.53 ± 0.05	0.070	0.60 ± 0.08	0.61 ± 0.06	0.944
JSA_sym_ [a.u.]	0.47 ± 0.04	0.37 ± 0.04	**0.022**	0.39 ± 0.06	0.32 ± 0.04	0.110
JSA_dia_ [a.u.]	0.03 ± 0.01	0.05 ± 0.01	**0.016**	0.06 ± 0.02	0.07 ± 0.01	0.850

	Mxa < 0 (n = 15)			Mxa ≥ 0 (n = 12)		
	Baseline	Hypocapnia	p value	Baseline	Hypocapnia	p value
AMP [mmHg]	2.46 ± 0.5	1.77 ± 0.5	0.355	1.35 ± 0.23	1.01 ± 0.21	0.294
AmpCBFV [cm/s]	21.97 ± 2	18.53 ± 2.00	0.145	20.76 ± 3.0	19.15 ± 2.0	0.645
RAMP [a.u.]	0.71 ± 0.03	0.59 ± 0.05	0.070	0.57 ± 0.08	0.54 ± 0.06	0.730
JSA_sym_ [a.u.]	0.44 ± 0.04	0.37 ± 0.04	0.090	0.43 ± 0.06	0.32 ± 0.04	**0.026**
JSA_dia_ [a.u.]	0.03 ± 0.01	0.05 ± 0.01	0.11	0.06 ± 0.02	0.07 ± 0.01	0.345

The data are presented as the means with standard errors. Significant differences are marked in bold

ICP, intracranial pressure; AMP, intracranial pressure pulse amplitude; AmpCBFV, pulse amplitude of cerebral blood flow velocity; RAMP, correlation between AmpCBFV and AMP; RAP, compensatory reserve; PRx, pressure reactivity index; JSA_sym_, joint symbolic analysis (symmetrical); JSA_dia_, joint symbolic analysis (diagonal), Mxa, mean velocity index

## Discussion

In this study, we investigated the relationship between the vascular and parenchymal intracranial compartments by analysing AmpCBFV and AMP during mild hypocapnia manoeuvres in TBI patients. Our findings revealed that mean AmpCBFV and AMP were not significantly correlated, either at baseline or during hypocapnia, when analysed using single average values *per* patient and a correlation coefficient *per* cohort. However, when the relationship between AmpCBFV and AMP were analysed individually and continuously, using a moving-window correlation index, named RAMP, significant changes were observed between baseline and hypocapnia. Additionally, using the nonlinear method of JSA, we found that during hypocapnia, there was a shift in the AmpCBFV-AMP relationship from the symmetry to an opposite direction pattern. Moreover, these changes may be more pronounced in patients with normal ICP and cerebral autoregulation but may be suppressed in patients with impaired autoregulation.

Intracranial vessels respond promptly to changes in CPP to maintain adequate CBF via a cerebral autoregulatory mechanism. The cerebral vasculature is particularly sensitive to chemical stimuli, such as O_2_ and CO_2_ tension in arterial blood. As all of those cerebral circulatory processes take place in a closed space of the skull, any modification of vessel diameter may affect the ICP through changes in CBV. Moreover, the ICP, CBF and CBV are affected by noncirculatory factors, such as craniospinal elasticity and CSF dynamics [31]. These mechanisms interact in complex nonlinear ways, which, in addition to the many factors influencing them, complicate the analysis of cerebrovascular regulation. As simple qualitative terms may be not sufficient to describe dependencies in cerebral circulatory processes, various mathematical models and indices have been proposed in recent years [28, 29, 31–34]. Moreover, in addition to general models of intracranial dependencies, several physiological parameters and indices have been evaluated to measure hemodynamic changes during hyperventilation and/or hypocapnia in TBI patients [20, 30].

Moderate hyperventilation, which leads to temporary, mild hypocapnia, is one of the simplest treatments for the management of intracranial hypertension. The response to changes in partial CO_2_ depends on the reactivity of the brain’s vessels, which could be impaired after injury, and the compliance of the brain [20]. Mild hypocapnia leads to a reduction in ICP and an increase in CSF compliance, which is counterbalanced by a decrease in cerebral blood flow and cerebral arterial compliance [35]. The physiological mechanisms involved in these effects involve a direct relationship between an increase in cerebrovascular tone and an increase in cerebrovascular smooth muscle calcium levels [36, 37].

In this study, we investigate the utility of two approaches: the linear, correlation-coefficient-based index (RAMP) and the nonlinear JSA method. In RAMP index the rationale for averaging AmpCBFV and AMP waveforms over 10 s is similar to that in the PRx or Mxa definition, as only slow waves, with frequencies lower than 0.05 Hz, can carry information about the autoregulation process [38]. Instead of determining the correlation coefficient for the means of AmpCBFV and AMP in a cohort of patients, we proposed an index, which dynamically allows us to describe the interdependency between those two neuroparameters.

The JSA analysis is adapted from studies on cardiovascular coupling and cardiorespiratory interactions and has been repurposed in this study to investigate intracranial dynamics. It is most commonly used in cardiological research to explore fast-changing dynamics [39, 40]. When analysing heart rate and systolic blood pressure dynamics, symbolic representations of beat-to-beat changes in the R-R interval and systolic arterial pressure provide an effective embedding of baroreflex-related R-R interval dynamics. JSD_sym_ representing symmetric word types, indicating the relative frequency of baroreflex-like word types, while JSD_diam_ represents the relative frequency of patterns opposed to baroreflex behaviour [19]. JSA is based on assessing the probability of joint patterns, expressed as a percentage, and describes coordinated behaviours between two time-series (in our study, AmpCBFV and AMP). Nonlinear symbolic dynamics is a powerful approach that involves coarse-graining observed time series into sequences of symbols (‘words’). Although detailed information is lost in the process, significant patterns emerge that can be used to quantify system dynamics [19]. The utility of a nonlinear approach for analysing AmpCBFV-AMP is further justified, as this relationship is inherently nonlinear [31]. Both JSA-derived metrics (JSD_sym_ and JSD_diam_) provide information about synchronization or interaction between parenchymal and vascular compartment (see Fig. 3). However, further studies are needed to assess whether this approach can be directly applied to the interpretation of correlation-based RAMP index.

We found that the relationships between AmpCBFV and AMP at baseline and during the hypocapnia plateau phase may differ in terms of the state of cerebral autoregulation and mean ICP level. In patients with preserved cerebral autoregulatory mechanisms (assessed using PRx) and in whom the ICP is not elevated, JSA_sym_ significantly decreases during hypocapnia. These changes were not observed in patients with an ICP above 20 mmHg or with impaired cerebral autoregulation (using PRx), which suggested that this interdependency may be suppressed. However, when the Mxa index of cerebral autoregulation was used we found that during impaired cerebral autoregulation, the synchronisation between AMP and AmpCBFV significantly decreased reflected as JSA_sym_. Notably, only the JSA, but not linear metrics, allows us to observe those differences. The response of cerebral vessels to stimuli from CO_2_ concentrations depends on the reactivity of the brain’s vessels; however, further investigations on the pathophysiological mechanism of these findings are needed. Moreover, our results indicated that PRx and Mxa should not be equated, especially during manoeuvre when cerebral blood volume occurred.

As technology advances, the management of TBI patients is continually improved through novel methods of waveform analysis. Neuromonitoring-based indices, introduced as mathematical concept, such as optimal CPP (CPP_opt_) [41, 42] and the pulse shape index (PSI) [27, 43] have shown promise as tools for assessing neurocritical care patients’ condition. Both the linear RAMP index and non-linear JSA algorithm may serve as valuable tools in ICU bedside monitoring, providing synchronised information about two intracranial compartments: vascular and intraparenchymal. This continuous monitoring may offer clinically relevant early warnings of brain deterioration, unlike imaging methods that only provide a ‘snapshot’ of brain condition. Further validation with computed tomography or magnetic resonance imaging is needed to evaluate the utility of RAMP and JSA-derived metrics in identifying midline shift or mass lesions following TBI.

### Limitations

This study has several limitations. For our analysis, we used data collected from patients who were hospitalised several years ago. Therefore, the retrospective nature of the data and advancements in treatment protocols may affect the generalizability of the findings to current clinical practice. We applied thresholds for ICP as 20 mmHg, PRx and Mxa as 0 to provide more balanced subgroups; however, the results should be confirmed for thresholds for ICP ≥ 22 mmHg [44] and PRx > 0.3 and Mxa > 0.3 [45]. Considering the binary symbolisation scheme, words of length three result in 64 different word types. Although word lengths *k* = 3 have been predominantly used in the literature [39], it is a pragmatic solution rather than a true reconstruction of the system's trajectory [19] and was applied in cardiovascular dynamics studies, but not for neuromonitoring parameters. Although we assessed patients from a homogeneous group, the impact of the baseline condition of cerebral autoregulation may influence the results observed during the hypocapnia manoeuvre. Further research with more recent data and larger cohorts is needed to validate and refine our conclusions.

## Conclusions

Our results indicate that while the mean AMP and AmpCBFV were not significantly correlated, at baseline or during hypocapnia, significant changes were observed when the RAMP index and JSA were analysed dynamically. Further validation in a larger cohort is needed.

## Data Availability

The data supporting this study will be made available upon reasonable request.
